# Photogrammetric and LiDAR Scanning with iPhone 13 Pro: Accuracy, Precision and Field Application on Hazelnut Trees

**DOI:** 10.3390/s25185629

**Published:** 2025-09-09

**Authors:** Elèna Grobler, Giuseppe Celano

**Affiliations:** 1 School in Agricultural, Forest and Food Sciences, University of Basilicata, Via dell’Ateneo Lucano 10, 85100 Potenza, Italy; 2 Course of Agriculture, Pharmacy Department, University of Salerno, Via Giovanni Paolo II, 132, 84084 Fisciano, Italy; gcelano@unisa.it

**Keywords:** smartphone-based sensing, precision agriculture, biometric parameters, 3D point clouds, tree modelling, digital phenotyping, low-cost sensing, *Corylus avellana* L.

## Abstract

Accurate estimation of tree structural and morphological parameters is essential in precision fruit farming, supporting optimised irrigation management, biomass estimation and carbon stock assessment. While traditional field-based measurements remain widely used, they are often time-consuming and subject to operator-induced errors. In recent years, Terrestrial Laser Scanning (TLS) and UAV-based photogrammetry have been successfully employed to generate high-resolution 3D reconstructions of plants; however, their cost and operational constraints limit their scalability in routine field applications. This study investigates the performances of a low-cost, consumer-grade device—the iPhone 13 Pro equipped with an integrated LiDAR sensor and RGB camera—for 3D scanning of fruit tree structures. Cylindrical targets with known geometric dimensions were scanned using both the LiDAR and photogrammetric (Photo) modes of the Polycam© application, with accuracy and precision assessed by comparing extracted measurements to reference values. Field applicability was also tested on hazelnut trees, assessing height, stem diameter and leaf area: the Photo mode delivered the highest accuracy (systematic error of 0.007 m and R^2^ = 0.99) and strong agreement with manual leaf measurements (R^2^ = 0.93). These results demonstrate that smartphone-based 3D scanning can provide a practical, low-cost approach for structural characterisation in fruit orchards, supporting more efficient crop monitoring.

## 1. Introduction

In the current century, the agricultural sector is facing increasing global challenges, such as climate change—towards which it contributes significantly—and the progressive reduction in essential natural resources such as water and soil. At the same time, the growing global population is driving an increasing demand for food, emphasising the urgency of adopting more sustainable crop management strategies from environmental, economic and social standpoints. Among cultivated systems, perennial woody crops—particularly fruit trees—play a dual role, contributing not only to food production but also to long-term carbon sequestration through the accumulation of structural biomass. The estimation of aboveground biomass, in this context, becomes an essential operation to support both agronomic management (irrigation, pruning and fertilisation) and the quantification of carbon storage.

In forestry, aboveground biomass has traditionally been estimated through non-destructive methods such as allometric equations [1,2,3], mathematical models that relate the dry mass of a tree to measurable variables such as diameter and height. Although generalised formulations have been developed for different forest types and species [4], these equations are generally site-specific and can hardly capture the geometric complexity and morphological variability of real plant structures. In recent years, advanced sensing technologies such as photogrammetry and laser scanning have been increasingly adopted in forest environments for structural analysis of vegetation. These methods, implemented through both terrestrial [5,6] and airborne platforms [7,8,9], have demonstrated a strong potential for generating detailed three-dimensional reconstructions of canopy architecture, proving particularly effective for volumetric analysis and the estimation of aboveground biomass [10].

Building upon these advances, similar approaches have subsequently been applied in agricultural and fruit tree systems, where the integration of 2D and 3D LiDAR technology and drone-based photogrammetry has supported the acquisition of high-resolution structural data of individual plants and entire orchards [11,12,13,14]. Nevertheless, the widespread adoption of these systems in operational settings remains limited, primarily due to their cost, technical complexity and the requirement for specialised expertise in data acquisition and processing. In this context, the emergence of consumer-grade devices equipped with integrated sensors, such as smartphones featuring LiDAR and high-resolution RGB cameras, represents a promising alternative. These devices rely on augmented reality (AR) frameworks, which integrate information from multiple onboard sensors to create a consistent spatial representation of the surrounding environment. Two main approaches are available: LiDAR-based acquisition, where an integrated laser scanner emits infrared pulses and measures the return time to generate depth maps, which are fused with RGB imagery to produce a georeferenced point cloud in real time; Photogrammetry, a methodology that reconstructs 3D geometry from a set of overlapping RGB images using feature-matching and triangulation algorithms, yielding high-resolution point clouds. The diffusion of AR frameworks in mobile devices has made both methods accessible through user-friendly applications.

Recent studies have demonstrated the technical feasibility of using such devices for various forest inventory tasks, particularly diameter at breast height (DBH) estimation [15,16], small-scale forest measurements [17], using both iPhones and iPads equipped with LiDAR sensor [18,19], and improvement to UAV-derived canopy height models [20,21]. Beyond forest application, low-cost scanning systems have been tested in urban environments, including the structural analysis of isolated olive trees [22] and in the context of urban forest mapping [23,24]. In the agricultural domain, their use has mainly focused on fruit yield estimation, with applications reported in vineyards [25], citrus orchards [26] and kiwifruit plantations [27], and leaf measurements [28]. However current evidence remains limited for fruit tree systems: while some applications have been reported, they have mostly addressed fruit yield estimation or specific morphometric traits, without extending to a comprehensive structural characterisation of trees. In contrast, most studies have been carried out in forest and urban contexts, which differ substantially from fruit orchards in terms of tree size, planting density and management practices such as pruning. This highlights a clear scientific gap regarding the applicability and accuracy of consumer-grade scanning devices for structural analysis in fruit tree systems, particularly with respect to the estimation of aboveground biomass and associated carbon sequestration.

The present study aims to evaluate the performance of a consumer-grade device—the iPhone 13 Pro, equipped with an integrated LiDAR sensor and RGB camera—for the three-dimensional reconstruction and structural analysis of fruit trees components. The investigation followed a multi-step approach to assess both accuracy and practical feasibility under controlled and field conditions, including the estimation of morphometric traits of individual leaves. Particular emphasis was placed on the leaf area parameter, given its strong agronomic relevance as an indicator of photosynthetic capacity and transpiration dynamics. It was hypothesised that the two acquisition modes would show differences in accuracy and repeatability when applied to both controlled and field conditions, and that at least one of them would provide measurements with sufficient precision to enable reliable estimation of structural and morphological traits in fruit tree systems.

## 2. Materials and Methods

### 2.1. Experimental Setup and Acquisition

**Figure 1:**
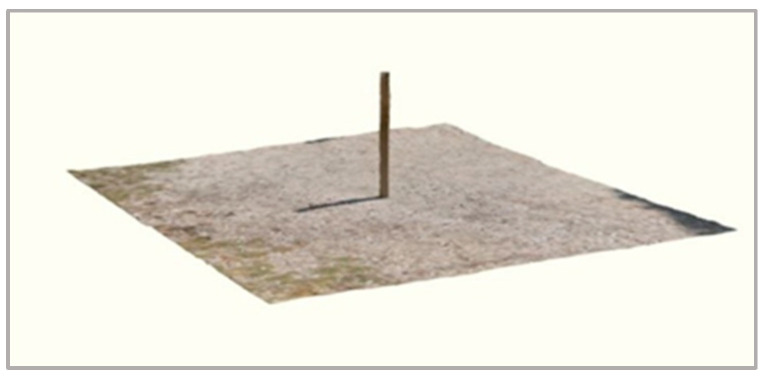
Example of a 3D reconstruction of a single cylindrical target acquired using the Polycam© application.

The experimental protocol was designed to assess the dimensional measurement capabilities of the iPhone 13 Pro’s LiDAR (Apple Inc., Cupertino, CA, USA), sensor under standardised conditions, with specific reference to applications in structural modelling of fruit tree components. The device running on iOS 15, includes a suite of onboard sensors: a proximity sensor, the True Depth system and a class 1 LiDAR unit. The LiDAR is based on a Direct Time-of-Flight (dToF) system composed of a VCSEL (Vertical-Cavity-Surface-Emitting Laser) emitter and a SPAD NIR CMOS (Single Photon Avalanche Diode Near-Infrared Complementary Metal-Oxide Semiconductor) receiver. Combined with gyroscope, magnetometer and RGB cameras, these components enable three-dimensional reconstructions in the form of point clouds. The iPhone 13 Pro was selected as it represents a low-cost and portable alternative to conventional terrestrial laser scanning systems, which, although highly accurate, are considerably more expensive; in addition, it integrates both LiDAR unit and high-resolution RGB camera within the same consumer-grade platform, enabling the acquisition of LiDAR-based and photogrammetric dataset using identical optical and processing conditions. The model was also chosen for its wide availability and its documented use in previous studies on forestry and urban vegetation studies [15,16,17,18]. No additional smartphones or tablets were tested, as the aim was to perform an in-depth evaluation of a single representative device for orchard applications. To simulate the geometry of aboveground tree structures, a total of 31 cylindrical models (pipes) with pre-measured diameters and heights were employed (Figure 1). Reference measurements were obtained manually using a digital calliper (resolution 0.01 mm) and a measuring tape. Acquisitions were performed in an open flat outdoor environment under clear spring weather conditions, characterised by full sunlight, no cloud cover and absence of wind. These stable environmental conditions minimised potential interference from shadows and reflections, ensuring acquisition stability and reproducibility.

The scanning procedure was conducted using the Polycam© app v.2.2.10 (Polycam inc., Staple House, Bedfordshire, UK), selected for its compatibility with both LiDAR and image-based 3D reconstruction workflows and its ability to export point cloud data in standard formats.

The application provides two acquisition modes:

- Photo mode, in which multiple images are captured around the object;
- LiDAR mode, where a continuous video scan is performed.

Both acquisition modes require a preprocessing step before data export: in photo mode point cloud reconstruction is cloud-based, and the processed output is downloaded to the device; in LiDAR mode preprocessing is performed locally on the smartphone, with user-defined voxel size settings. Point cloud data were processed using CloudCompare (v.2.12.4, EDF R&D, Paris, France) for spatial measurements.

### 2.2. Accuracy and Precision Tests

A preliminary experimental phase was conducted using eight cylindrical models with known geometric dimensions. For each acquisition mode, scans were performed at four standardised radial distances (1.0 m, 1.5 m, 2.0 m, 3.0 m), maintaining a constant device height of 1.5 m above ground level; each distance-mode configuration was repeated three times. The acquisition distances were selected to represent typical orchard conditions: shorter distances enabled the capture of fine details, whereas increasing the distance reproduced situations where canopy size, planting layout or operational constraints limit proximity to the target.

In LiDAR mode, supplementary acquisitions were also performed by varying the voxel resolution parameter (low, medium, high), as provided by the application settings, which defines the size of the three-dimensional units used to discretise the point cloud.

Accuracy was assessed separately for diameter and height, by calculating the mean deviation from the reference value and the standard deviation of that mean deviation. In addition, a global evaluation was conducted on all data aggregated by acquisition mode. Precision was evaluated by analysing the three repeated measurements obtained for each experimental configuration, defined by acquisition mode, scanning distance and target. For each configuration, the mean, standard deviation and relative standard deviation (RSD) were calculated to quantify measurement repeatability. In a second phase, all repeated measurements associated with each individual target were aggregated across distances and modes: this allowed the computation of target-specific RSD values, providing an overview of how measurement consistency varied with object size. The resulting RSD values were then plotted in relation to the reference dimensions of the targets to investigate precision trends across different scales.

**Figure 2:**
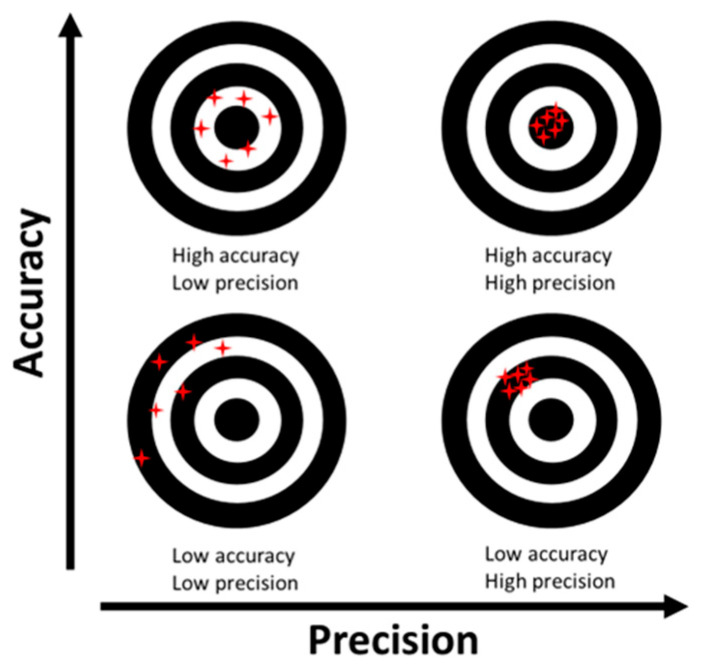
Representation of the four possible combinations of accuracy and precision in measurement systems. Each red star represents a repeated measurement; the centre of the target indicates the true value.

To assess the relationship between accuracy and precision, bivariate scatter plots were constructed, where each point represents a measurement pair. This classification enabled the identification of measurement categories: accurate and precise, accurate but imprecise, imprecise but accurate, and both imprecise and inaccurate, as reported in Figure 2.

Finally, to complement the assessment of accuracy and precision, a non-parametric Wilcoxon signed-rank test was performed on paired diameter and height measurements from LiDAR and Photo modes, as the data did not conform to the assumption of normality. The statistical analysis was conducted in R software (v 4.4.1; R Core Team, Vienna, Austria) using the wilcox.test() function from the stats package to determine the existence of statistically significant differences between the two acquisition methods.

### 2.3. Extended Dataset Analysis and Validation

Based on the findings of the preliminary phase, a set of 31 cylindrical targets was acquired entirely in Photo mode, at a standardised scanning distance of 1.5 m, to capture fine-scale details and ensuring full target coverage. Once processed, point clouds were analysed in CloudCompare to extract target height and diameter. Dimensional variables were analysed individually to investigate potential correlations. The full dataset was randomly divided into two subsets: a calibration set (*n* = 20), and a validation set (*n* = 11), to enable independent assessment of the instrument’s reliability.

### 2.4. Study Site

**Figure 3:**
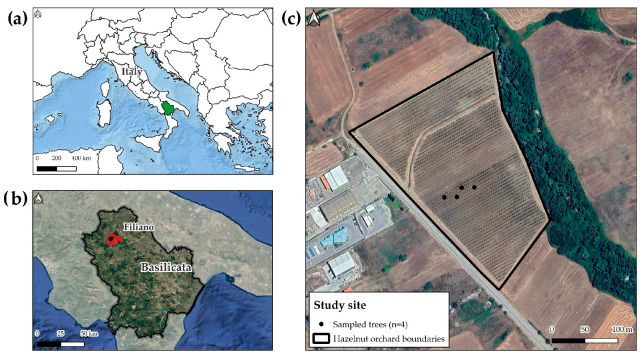
Geographical location of the study area. (**a**) Position of the Basilicata region within Italy (highlighted in green); (**b**) Location of the municipality of Filiano (Potenza) highlighted within the regional context (red), with the hazelnut orchard marked by a black dot; (**c**) Detail of the experimental hazelnut orchard selected for the trial.

The field application was conducted in a commercial hazelnut orchard located in Filiano (Potenza, Basilicata, Southern Italy; 40°49′50.61″ N, 15°42′13.07″ E), at an elevation of 480 m above sea level, with an average slope of 2% and a north-east orientation (Figure 3). The orchard covers a total surface of 4.9 ha, with a 4 × 5 m planting layout. The soil, classified as sandy-clay loam (Cambisols—Regosols), shows a neutral to slightly acidic pH (6.8), with a good organic matter content (3%). The area has a Mediterranean climate with continental influences, characterised by cold winters and dry summers. Trees are trained as multi-stemmed bushes, a widely adopted system in the regional production area, and the orchard is managed under conventional practices including mechanical weed control, periodic pruning and drip irrigation system. The site was selected for its homogeneous varietal composition, which minimised confounding effects of genetic or site variability.

### 2.5. First Application to Fruit Trees

**Figure 4:**
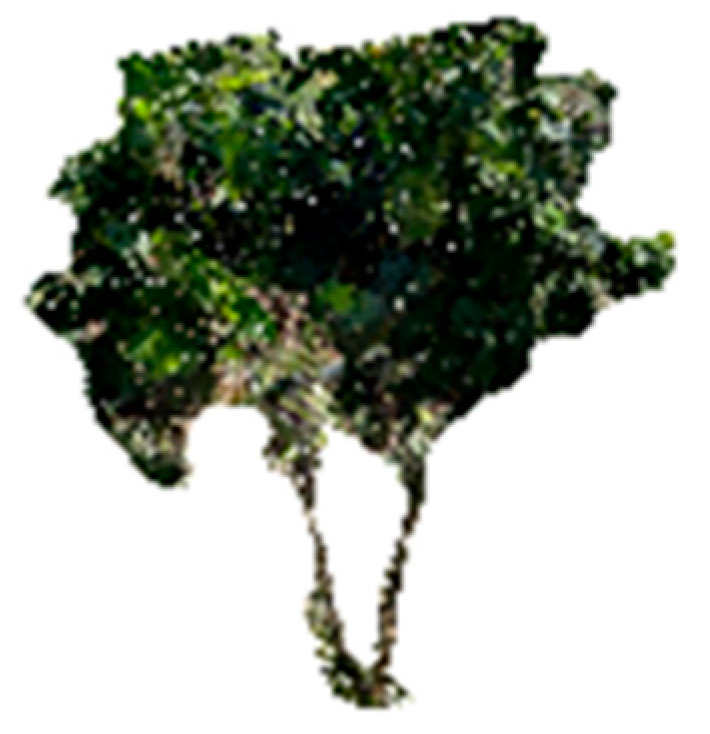
Point cloud of a *Corylus avellana* L. tree (cv. Tonda di Giffoni), generated through smartphone-based scanning at a fixed distance of 1.5 m and processed in CloudCompare. The reconstruction highlights the two distinct main stems, typical of the multi-stemmed bush training system.

To assess the feasibility of smartphone-based scanning in real-world horticultural conditions, a preliminary application was conducted on hazelnut trees (*Corylus avellana* L.) of the cultivar Tonda di Giffoni: this variety, widely recognised as one of the most representative in the Italian hazelnut sector, was selected as the focus of the trial given its presence in the experimental site. A subset of 4-year-old trees, trained as multi-stemmed bush with two main stems per plant (Figure 4), was selected for this preliminary assessment: this choice allowed the assessment of the methodology while ensuring homogeneity of age and structures. The trial was designed with the specific purpose of evaluating the feasibility and reproducibility of the methodology under orchard conditions, rather than to provide exhaustive biometric data. Scanning was performed using the Polycam© application in Photo mode, selected for its higher accuracy in previous tests. Each tree was scanned maintaining a constant distance of 1.5 m from both the tree and the ground level, to optimise image overlap and ensure complete coverage of the structures: the resulting 3D point clouds, each consisting of an average of 559,402 points, were exported in LAS format and processed in CloudCompare. Geometrical attributes, such as total height and diameter of the stems at 0.30 m height, were calculated.

### 2.6. Estimation of Leaf Area from 3D Point Clouds

**Figure 5:**
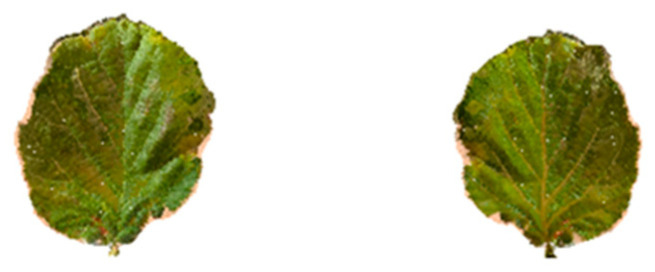
Front and back views of a 3D model of a *Corylus avellana* L. leaf acquired using the Photo mode of the Polycam© application on smartphone.

A complementary analysis was carried out to estimate the leaf area of *Corylus* avellana L. using a smartphone-based 3D scanning approach. Leaf samples were collected from the same 4-year-old orchard described above: a total of 20 mature, fully expanded and undamaged leaves were collected, then positioned vertically on a rigid support placed on a flat surface to ensure stability during acquisition. Each sample was scanned using Polycam ©application in Photo mode, maintaining 0.5 m between the smartphone and the leaf (Figure 5). 

The acquired point clouds, consisting of an average of 552,036 points, were exported in LAS format and processed in CloudCompare software: leaf surface was isolated by manually defining Region of Interest (ROIs) to exclude the petiole and any residual background points. Manual approach was preferred over automatic ROI selection based on colour thresholds, as the latter proved less precise due to the irregular margins of the hazelnut leaves. The isolated point cloud was then used to compute surface area using the 2.5D surface tool of the software. To assess the reliability of this method, results were compared with ImageJ v 1.54 (NIH, Bethesda, MD, USA) measurements: for this method, each leaf was photographed alongside a metric reference scale, images were processed by setting the scale, converting to 8-bit format, and adjusting threshold to isolate the leaf shape. Regions of interest (ROIs) were then manually selected and added to the ROI manager, which automatically returned the area values for each leaf.

## 3. Results

### 3.1. Accuracy Assessment

#### 3.1.1. Individual Variables

The accuracy of measurements was evaluated separately for diameter and height under both acquisition modes. In the LiDAR setting, supplementary test with the three voxel resolution levels available in the application produced point clouds of equivalent density, yielding identical measurement values: voxel resolution was therefore not considered further. For each combination, the mean deviation from the reference value (*Δ*), the associated standard deviation (*±σ*), and the normalised relative error (*Δ/x*) were calculated, where x represents the true value of the measured dimension. Results are reported in Table 1.

The analysis highlights substantial differences in performance between the two acquisition methods: LiDAR mode produced an underestimation in height measurements, whereas in Photo mode both variables were measured with high accuracy.

**Figure 6:**
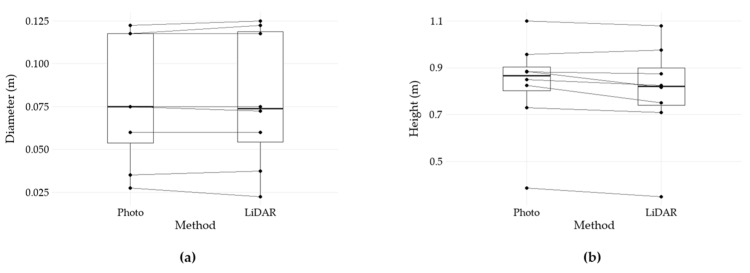
Boxplot of paired comparisons between Photo and LiDAR estimates. (**a**) Distribution of mean diameter values per target; (**b**) distribution of mean height values per target.

To complement the evaluation, a paired Wilcoxon signed-rank test was performed to compare LiDAR and Photo measurements separately for diameter and height (Figure 6). Results revealed no significant difference for diameter, whereas a statistically significant deviation was observed for height, with LiDAR returning lower values compared to Photo mode (Table 2).

#### 3.1.2. Aggregated Analysis

Aggregated results for diameter and height measurements (Table 3) show lower deviation and variability in Photo mode compared with LiDAR, highlighting its higher reliability when both dimensions are considered simultaneously.

### 3.2. Precision Assessment

#### 3.2.1. Precision by Target

Table 4 reports the relative standard deviation (RSD) values of diameter and height for each target, acquired in both LiDAR and Photo mode.

Photo mode provided more stable precision, particularly for height, where RSD values remained consistently low across all targets; in contrast LiDAR mode exhibited greater variability, especially in diameter measurements. For several targets, the height measured via LiDAR also showed increased dispersion, while Photo-mode remained below 0.05 in most cases.

#### 3.2.2. Precision by Distance

Average RSD values across all targets, grouped by acquisition distance, are summarised in Table 5.

A progressive reduction in RSD for height was observed in LiDAR mode, with the lowest variability recorded at a 3.0 m distance; Photo mode, in contrast, showed high repeatability at shorter distances, especially for diameter, but exhibited higher variability as distance increased. These results highlight the influence of acquisition range on precision and emphasise the superior stability of photogrammetric scans under close-range conditions.

### 3.3. Relationship Between Accuracy and Precision

**Figure 7:**
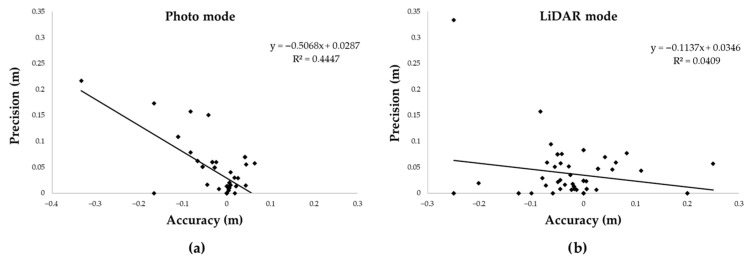
Relationship between accuracy and precision expressed as relative error and relative standard deviation. (**a**) In Photo mode a moderate positive correlation was observed; (**b**) In LiDAR mode, no significant association was found, suggesting independence between trueness and repeatability.

To explore potential dependencies between accuracy and precision, the values obtained for each target were plotted (Figure 7), pairing the relative error (Δ/x) with the relative standard deviation (RSD).

A moderate correlation was observed in Photo mode (R^2^ = 0.44), in contrast with no significant relationship found in LiDAR mode (R^2^ = 0.041), where accuracy and precision appeared independent. Although an R^2^ value of 0.44 represents only a moderate coefficient of determination, its interpretation within the context of the present study remains relevant: firstly, the correlation was positive, indicating that in Photo mode a reduction in relative error was associated with a reduction in relative standard deviation, thereby linking measurement trueness and repeatability. Secondly, the choice of Photo mode was based on an overall performance assessment, as it consistently yielded lower deviations, smaller relative errors and reduced measurement dispersion compared with LiDAR mode.

### 3.4. Regression Analysis on 31 Targets

Following the preliminary accuracy and precision assessment, Photo mode was selected for an extended evaluation on 31 cylindrical targets scanned at a fixed distance of 1.5 m. Measurements obtained from the point clouds were compared against the reference measurements values through linear regression analysis.

**Figure 8:**
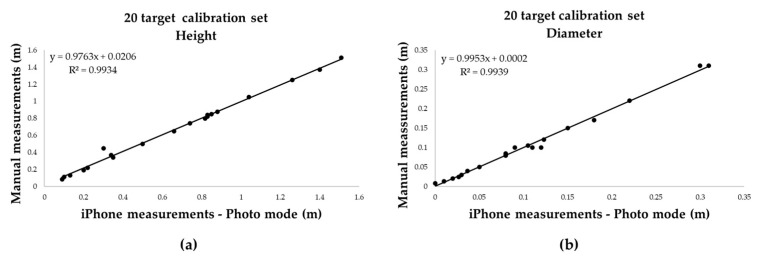
Linear regression between measured and true values on the 20—target calibration set; (**a**) height and (**b**) diameter.

The first dataset (20 targets) yielded a very strong relationship for both diameter and height (R^2^ =0.99) (Figure 8).

**Figure 9:**
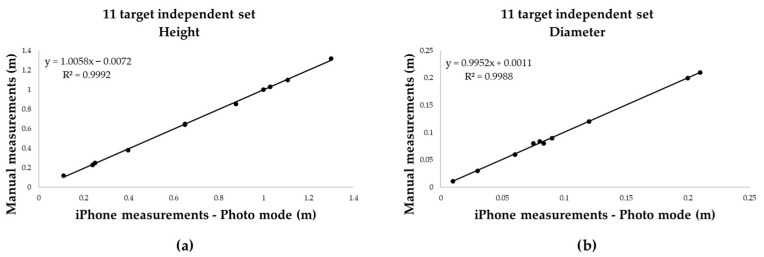
Validation of the regression models on the independent set (*n* = 11): (**a**) height; (**b**) diameter. The models maintained a high degree of concordance with reference values.

Model robustness was subsequently validated on an independent validation set (11 targets), which confirmed the high degree of concordance between measured and reference values (Figure 9).

### 3.5. Combined Variable Regression on 62 Targets

**Figure 10:**
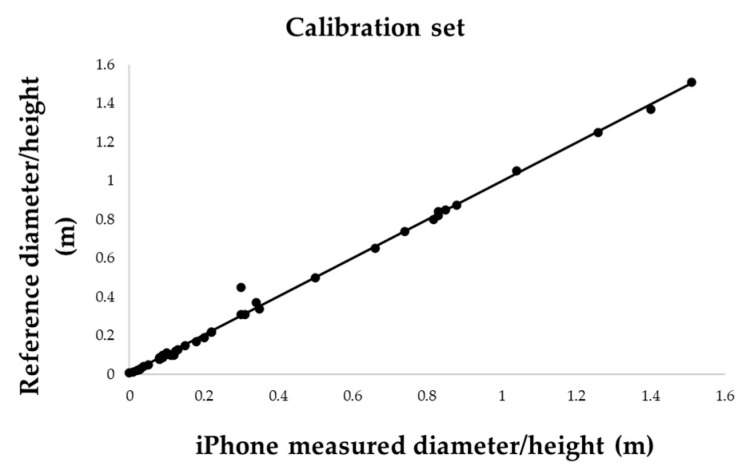
Combined regression model fitted on the calibration dataset (*n* = 40), including both diameter and height measurements. The regression equation (y = 0.9916*x* + 0.0056) shows an excellent fit with reference values (*R*^2^ = 0.99).

To assess the overall predictive performance of the system, diameter and height measurements were analysed jointly throughout a combined-variable regression. The full dataset (*n* = 62) was randomly divided into a training set of 40 samples and a test set of 22 samples. A linear regression model was fitted to the calibration set, where iPhone-derived measurements were regressed against manual reference values (Figure 10).

The resulting equation was:

(1) $$y=0.9916x+0.0056\;\mathrm{with}\;R^{2}=0.996$$

The model was then applied to the validation set by inputting the iPhone-measured values into the regression equation, generating predicted values. Specifically, for each target in the test set, the following formula was used:

(2) y=0.9916∗xiPhone+0.0056

**Figure 11:**
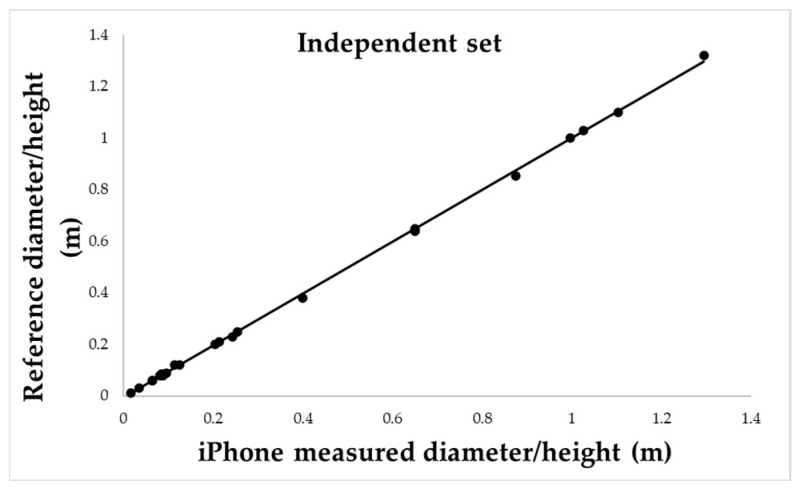
Validation of the combined regression model on the independent test dataset (*n* = 22). Predicted values were computed using the calibrated equation and compared with manual reference measurements, showing a high degree of concordance across both variables.

The predicted values were then compared to the manual reference measurements (Figure 11), yielding a high degree of concordance between predicted and reference dimensions. This combined regression analysis represents the final step on cylindrical targets, demonstrating that Photo mode provides accurate and consistent dimensional estimates.

### 3.6. Preliminary Application to Hazelnut Trees

The preliminary field application on four hazelnut trees (*Corylus avellana* L., cv. Tonda di Giffoni) demonstrated the feasibility of smartphone-based 3D scanning for structural trait estimation in fruit tree systems. Dimensional attributes, such as height and stem diameter at 0.30 m (Table 6), were successfully extracted from point clouds. Although minor occlusion and shadowing effects were observed, the Photo mode successfully captured the overall architecture of each tree, and the point clouds exhibited sufficient resolution to clearly identify the presence of two distinct stems per plant. These preliminary results indicate the potential of this method for rapid and non-destructive phenotypic data collection in field. Structural parameters obtained from smartphone-based models can support several agronomic applications, such as tree vigour monitoring, growth dynamic assessment and training system evaluation.

### 3.7. Leaf Area Estimation

**Figure 12:**
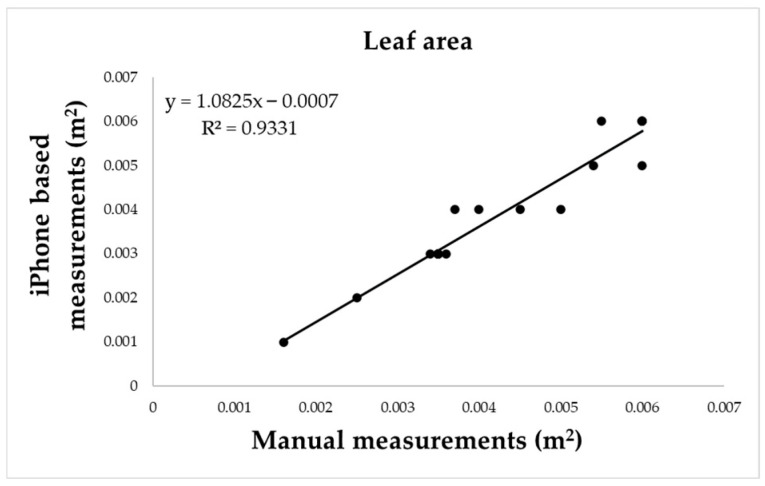
Linear regression between leaf area measurements obtained from smartphone-based 3D point clouds and manual measurements performed with ImageJ. The fitted model (*y* = 1.0825*x* − 0.0007, *R*^2^ = 0.93) indicates a strong correlation, with a slight but systematic overestimation of 3D-derived values.

Smartphone-based 3D scanning of hazelnut leaves produced point clouds with sufficient resolution to allow the extraction of surface measurements through the 2.5D tool in CloudCompare. Estimated leaf area values were compared to those obtained manually using ImageJ, which served as reference method. The regression analysis revealed a strong linear relationship between the two datasets (Figure 12), described by the equation:

(3) $$y\;=\;1.0825x\;-\;0.0007\;\mathrm{with}\;R^{2}=0.9331$$

While a slight overestimation was observed in the 3D-derived values, the deviation appeared systematic and consistent across the range of leaf sizes, the scatter of data points along the regression line remained contained, suggesting that the variability between the two methods is limited. These results suggest that smartphone-based 3D models can provide reliable estimates of leaf area, a key trait for assessing photosynthetic capacity, light interception and overall transpiration in orchard systems.

## 4. Discussion

### 4.1. Performance of Smartphone-Based Scanning

The results of this study confirm the technical feasibility of using a consumer-grade smartphone (iPhone 13 Pro) for 3D reconstruction of fruit tree structures, under both controlled and field conditions. The initial calibration experiment revealed a clear difference between the two acquisition modalities implemented in the Polycam© application: Photo mode consistently exhibited higher accuracy and precision compared to the integrated LiDAR sensor. Separate analysis for diameter and height showed that Photo mode achieved mean absolute deviations below 3 millimetres for both variables, and relative error less than 5%, indicating a high level of measurement fidelity. In contrast, LiDAR mode systematically underestimated height, with a mean deviation of—0.027 m and a relative error of—3.9%, demonstrating that its lower spatial resolution limits reliability for detailed structural measurements at short range. These differences were statistically supported by the paired Wilcoxon signed-rank test, which confirmed the absence of significant deviations for diameter but detected a systematic underestimation of height in LiDAR mode compared with Photo acquisition.

When considering aggregated measurements for both variables, the superiority of Photo mode was confirmed: it yielded a near-zero mean deviation (−0.001 m) and lower variability (σ = 0.016 m) compared to LiDAR (−0.013 m; σ = 0.024 m), confirming that the photogrammetric reconstruction outperformed LiDAR in terms of spatial density and measurement accuracy. Furthermore, the precision assessment across different targets and acquisition distances reinforced these conclusions: Photo mode maintained low relative standard deviations (RSDs), particularly for height where values remained consistently below 5% for all targets; LiDAR mode showed stable precision at larger distances, but its performance was more variable, especially for diameter where RSD values reached up to 19.6%. These results underscore that the performance of smartphone-integrated LiDAR is more sensitive to object geometry and surface reflectance than photogrammetry, especially under close-range conditions, highlighting a critical limitation for the application of smartphone-integrated LiDAR in heterogeneous orchard environments.

The analysis of the relationship between accuracy and precision provided further insights: a moderate correlation (R^2^ = 0.44) was observed in Photo mode, an absent relationship in LiDAR mode (R^2^ = 0.04). This behaviour confirms previous findings from in forest applications, which reported that smartphone-based LiDAR performed poorly when applied to young plantation trees, showing low accuracy and no consistent relationship between measurement variability and reliability [18]: such evidence suggests that the constraints of the sensor are inherent to the technology rather than solely dependent on the study context.

Building on the performance observed in controlled conditions, a larger dataset of 31 cylindrical targets was analysed to evaluate the robustness of Photo mode: regression models fitted on 20 targets yielded near-perfect linearity for both height and diameter (R^2^ = 0.9939), which was maintained when applied to an independent validation set of 11 targets. The combined variable regression using all 62 samples confirmed the strength of the model, with R^2^ = 0.996 and mean absolute error of just 0.007 m.

### 4.2. Application to Orchard Systems and Leaf Traits

When applied in an operational orchard context, the smartphone-based method proved capable of capturing the structural characteristics of multi-stemmed hazelnut trees. Height and trunk diameter were successfully extracted from 3D point clouds acquired in Photo mode at a fixed distance of 1.5 m, confirming the potential of this tool for tree-level inventory in perennial fruit crops. These results demonstrate the feasibility of transferring methodologies—already validated in urban forest applications such as DBH estimation and tree mapping [22,23]—to the context of commercial orchards, where the geometric complexity and growth architecture differ significantly from typical forest stands due to pruning and agronomic practices. Moreover, the outcomes suggest that consumer-grade 3D scanning may complement more advanced and costly technologies such as terrestrial laser scanning and UAV-based photogrammetry [20,21]. In addition to structural measurements, the study investigated the feasibility of estimating leaf area—a parameter of agronomic relevance due to its strong correlation with photosynthetic activity, transpiration dynamics and plant water status—through 3D reconstructions of leaves. Twenty mature hazelnut leaves were scanned under field conditions, and their surface area was calculated from the resulting point clouds using CloudCompare software. These values were then compared against reference measurements obtained through manual image-based analysis with ImageJ, a validated and widely adopted tool for morphometric evaluation in plant science [29]. The comparison yielded a high coefficient of determination (R^2^ = 0.9331), confirming the reliability of the 3D scanning approach for capturing fine-scale leaf morphology. These findings are consistent with the recent work of Bar-Sella et al. [28], who demonstrated the capability of the iPhone 13’s acquisition to precisely reconstruct individual maize leaves, and extend the methodology to a woody species under field conditions, showing that accuracy can be preserved despite higher variability introduced by environmental factors.

### 4.3. Methodological Strengths and Limitations

The present study offers a comprehensive evaluation of the capabilities and limitations of a consumer-grade device—specifically the iPhone 13 Pro—for the three-dimensional characterisation of fruit tree structures and leaf morphological traits. The choice of this device was motivated by its integration of both active (LiDAR) and passive (photogrammetry) 3D acquisition modes within a single platform, combined with the maturity and stability of its hardware-software ecosystem. Such configuration made it suitable for a systematic and reproducible performance assessment, minimising variability associated with device heterogeneity. Furthermore, the widespread adoption of iPhone models enhances the transferability and broader applicability of the results, as the technology is readily accessible to a large user base, facilitating replication of the approach and its potential integration into routine agronomic practices. Through a combination of calibration tests, field applications on hazelnut trees and fine-scale reconstruction, we demonstrated that this portable and accessible technology can produce accurate and reliable results under a variety of operational conditions. The results obtained under controlled conditions revealed that, while both LiDAR and photogrammetric acquisition modes can be used for 3D reconstruction, the Photo mode consistently outperformed LiDAR in terms of spatial fidelity, accuracy and repeatability. These findings are consistent with previous studies in forestry [18], yet the present work extends these observations to the agricultural context, where fruit trees morphology and the variability introduced by agronomic management impose additional challenges. Compared to the forest-based results of Tatsumi et al., [17] and Oikawa et al., [19], data suggest that photogrammetry can offer greater robustness in environments where object shape, leaf cover and lighting are less controlled and more variable. When applied in orchard, the morphological complexity introduced by pruning systems and varietal growth patterns, represents a significant barrier to the generalisation of allometric models from forestry to fruit production systems; nevertheless, the approach enabled retrieval of key structural parameters without the need for expensive equipment.

These tools could complement TLS systems [5,6] or airborne systems [11,12,13], particularly in contexts requiring high temporal resolution or rapid deployment across multiple sites. Furthermore, the capacity of the method to estimate leaf surface area in situ suggests that smartphone-based scanning could support a more integrative characterisation of plant traits: this is particularly relevant in fruit crops, where both canopy structure and leaf area are key determinants of productivity, water use and resource allocation. The ability to acquire such parameters rapidly and non-destructively opens opportunities for improved monitoring of plant status over time, including under different irrigation or pruning regimes.

Despite its strengths, several limitations emerged: the photogrammetric workflow, while highly accurate at short range, is inherently sensitive to environmental factors such as ambient lighting, operator movement and background clutter. These conditions, typical of field environments, may introduce inconsistencies in point cloud generation or processing. In contrast, LiDAR’s performance, although more stable under variable conditions, remains constrained by low point density and irregular spatial distribution, a limitation that in this study resulted in a systematic underestimation of height measurements on the calibration targets. This bias may become particularly critical when extrapolated to tree-level indices dependent on vertical extent, such as total height or height to diameter ratios. Similarly, in orchard conditions, occlusions from foliage, multi-stemmed structures and variable illumination further contribute to measurement uncertainty. Ensuring adequate angular coverage, performing repeated acquisitions under varying conditions, and adopting a standardised acquisition protocol would represent essential measures to secure reproducibility. Additional limitations deserve mention. The analysis did not stratify trees by DBH classes, since the orchard consisted of coetaneous individuals with highly uniform trunk size: while this design was suitable to test the feasibility of the approach in a controlled field setting, future applications should extend the validation to trees of different stem diameter, as this parameter may influence reconstruction accuracy. Operator variability also remains untested, an essential aspect for assessing robustness in routine agronomic contexts. Finally, scalability represents a limitation, as the tree-by-tree approach constrains applicability in large or high-density orchards. Within this framework, the most appropriate application of smartphone-based scanning is the rapid characterisation of individual trees or the ground-truthing of UAV and TLS products, whereas extensive, high-density surveys remain more consistent with dedicated platforms, although at the cost of greater economic and operational requirements.

### 4.4. Operational Relevance and Future Perspectives

From an operational standpoint, the simplicity and portability of smartphone-based scanning represent a major advantage in context where rapid assessment, minimal training and low cost are critical requirements. Practically, the method provides a non-destructive approach for the quantitative monitoring of tree structural parameters. These measurements can be incorporated into orchard management frameworks to support evidence-based decisions on pruning, irrigation and nutrient supply. The possibility of deploying the same device across multiple sites further enhances data comparability, facilitating the establishment of consistent time-series datasets and improving the assessment of plant responses to agronomic interventions such as fertilisation or irrigation scheduling.

Finally, this study lays the groundwork for further research on the integration of smartphone-derived 3D data into practical phenotyping workflows. Future efforts should focus on expanding validation across different species, environmental conditions and time points, to evaluate the method’s robustness and scalability in real horticultural systems. Although comparison across different smartphone models was beyond the scope of the work, addressing inter-model variability will be crucial to ensure methodological consistency and broader applicability. In addition, the assessment of operator-related variability is needed to establish reproducibility in routine monitoring, while scalability challenges may be addressed through the integration of smartphone-based approaches with complementary platforms such as UAVs and TLS, enabling multi-scale workflows that combine low-cost, high-frequency tree-level observations with spatially extensive surveys, enhancing both temporal resolution and spatial coverage.

## 5. Conclusions

This study assessed the accuracy and precision of a consumer-grade smartphone, the iPhone 13 Pro, for the three-dimensional data acquisition, providing a comparison between LiDAR and photogrammetric modes under controlled and field conditions. Controlled tests demonstrated that photogrammetry consistently outperformed LiDAR in terms of spatial fidelity and repeatability, while LiDAR showed a systematic underestimation of the height parameter. Beyond calibration, the workflow was tested on hazelnut trees, allowing retrieval of key structural parameters, and on leaves, where results showed strong correspondence with direct measurements.

These findings confirm the technical reliability of smartphone-based photogrammetry as a practical tool for monitoring in fruit systems. By enabling the extraction of dimensional traits with low-cost and portable equipment, the approach supports applications ranging from tree structural assessment to the estimation of leaf characteristics. The possibility of obtaining reliable three-dimensional data without specialised instruments broadens accessibility to digital technologies, fostering their adoption in precision fruit farming.

Nonetheless, some limitations should be acknowledged, including the sensitivity of photogrammetry to environmental factors, the requirement for tree-by-tree scanning, which limits scalability, and the influence of operator variability. Further research should extend validation across different tree sizes, species and environments, and evaluate multiple smartphone or portable device models, to strengthen robustness and enable broader adoption in orchard management.

In conclusion, by demonstrating accurate structural and morphological data acquisition with a widely accessible device, this work advances the development of low-cost and field-ready technologies for digital fruticulture. While challenges remain in terms of scalability and inter-operator consistency, these findings lay the groundwork for broader integration of consumer-grade devices into digital fruticulture, bridging the gap between research applications and routine orchard management.

## Figures and Tables

**Table 1:** Accuracy metrics for diameter and height measured using LiDAR and Photo modes. *TA* = True average value (m); *Δ* = absolute mean deviation (m); *Δ/x* = relative error normalised by reference value; *OA* = Overall Accuracy (%).

Variable	Mode	*TA* (m)	*Δ* (m)	*±σ* (m)	*Δ/x*	*±σ (Δ/x)*	*OA* (%)
Diameter	LiDAR	0.10	0.00	0.01	−0.01	0.12	99
Height		0.67	−0.03	0.03	−0.04	0.04	96
Diameter	Photo	0.10	−0.00	0.00	−0.05	0.08	97
Height		0.67	0.00	0.02	0.00	0.03	100

**Table 2:** Paired Wilcoxon signed-rank test results for Photo and LiDAR measurements. Reported values include the test statistic (*V*), *p*-value and 95% confidence interval (*CI*) for the difference between paired measurements. Diameter shows no significant difference, while height presents a significant deviation.

	*p*-Value	*V*	95% *CI*	
Height (m)	0.0006	24.5000	−0.0600	−0.0300
Diameter (m)	0.2825	32	−0.01	0.01

**Table 3:** Aggregated accuracy metrics for LiDAR and Photo modes (diameter + height). *Δ* = absolute mean deviation (m); *Δ/x* = relative error.

Mode	*Δ* (m)	*±σ* (m)	*Δ/x*	*±σ (Δ/x)*
LiDAR	−0.01	0.02	−0.02	0.12
Photo	−0.00	0.02	−0.02	0.07

**Table 4:** Relative standard deviation (RSD) and corresponding precision (P) of diameter and height measurements by target and acquisition mode. Precision values are expressed as percentages.

Variable			Diameter	Height	Diameter	Height
Mode			LiDAR		Photo	
Target	1	RSD	0.20	0.03	0.20	0.02
		P	80.43	96.66	80.43	98.39
	2	RSD	0.15	0.03	0.09	0.04
		P	85.44	96.95	90.84	95.62
	3	RSD	0.05	0.02	0.07	0.02
		P	94.86	98.19	93.13	97.70
	4	RSD	0.08	0.08	0.04	0.03
		P	91.76	91.92	95.55	97.28
	5	RSD	0.02	0.01	0.09	0.03
		P	98.22	98.90	90.78	96.99
	6	RSD	0.07	0.06	0.05	0.05
		P	92.98	94.37	95.23	95.36
	7	RSD	0.11	0.04	0.05	0.00
		P	88.61	96.42	94.91	100.00

**Table 5:** Mean relative standard deviation (RSD) by acquisition distance and mode.

		Distance (m)			
Variable	Mode	1.0	1.5	2.0	3.0
Diameter	LiDAR	0.04	0.07	0.08	0.07
Height		0.02	0.03	0.02	0.01
Diameter	Photo	0.01	0.06	0.08	0.09
Height		0.01	0.01	0.03	0.02

**Table 6:** Structural parameters of hazelnut trees (*Corylus avellana* L., cv Tonda di Giffoni) acquired using smartphone-based scanning. Total height and stems diameter at 0.30 m were extracted from point cloud models processed in CloudCompare.

Tree	Height (m)	Diameter 1 (m)	Diameter 2 (m)
Tree1	1.61	0.03	0.04
Tree2	1.98	0.02	0.04
Tree3	1.49	0.03	0.03
Tree4	1.89	0.08	0.02

## Data Availability

The data that support the findings of this study are available upon reasonable request from the corresponding author.
